# The Medical and Surgical Reporter

**Published:** 1858-11

**Authors:** 


THE MEDICAL AND SURGICAL REPORTER, which has heretofore appeared as a monthly, was presented to us on the 6th of October under a new garb, and will henceforth be issued as a weekly journal, under the editorial management of Drs. Butler and Atkinson. This, with the exception of the *Boston Medical and Surgical Journal*, is the only weekly in this country. London alone supports three weekly journals, and we may therefore assume that under careful management, and with the assistance of the profession, the "Reporter" will take a creditable stand among our many periodicals. We wish the editors the best success in their new enterprise.

